# Applying psychological theories to evidence-based clinical practice: Identifying factors predictive of managing upper respiratory tract infections without antibiotics

**DOI:** 10.1186/1748-5908-2-26

**Published:** 2007-08-03

**Authors:** Martin P Eccles, Jeremy M Grimshaw, Marie Johnston, Nick Steen, Nigel B Pitts, Ruth Thomas, Elizabeth Glidewell, Graeme Maclennan, Debbie Bonetti, Anne Walker

**Affiliations:** 1 Institute of Health and Society, Newcastle University, Newcastle upon Tyne, UK; 2 Clinical Epidemiology Programme, Ottawa Health Research Institute and Department of Medicine, University of Ottawa, Ottawa, Canada; 3 School of Psychology, University of Aberdeen, Aberdeen, UK; 4 Dental Health Services Research Unit, University of Dundee, Dundee, UK; 5 Health Services Research Unit, University of Aberdeen, Aberdeen, UK

## Abstract

**Background:**

Psychological models can be used to understand and predict behaviour in a wide range of settings. However, they have not been consistently applied to health professional behaviours, and the contribution of differing theories is not clear. The aim of this study was to explore the usefulness of a range of psychological theories to predict health professional behaviour relating to management of upper respiratory tract infections (URTIs) without antibiotics.

**Methods:**

Psychological measures were collected by postal questionnaire survey from a random sample of general practitioners (GPs) in Scotland. The outcome measures were clinical behaviour (using antibiotic prescription rates as a proxy indicator), behavioural simulation (scenario-based decisions to managing URTI with or without antibiotics) and behavioural intention (general intention to managing URTI without antibiotics). Explanatory variables were the constructs within the following theories: Theory of Planned Behaviour (TPB), Social Cognitive Theory (SCT), Common Sense Self-Regulation Model (CS-SRM), Operant Learning Theory (OLT), Implementation Intention (II), Stage Model (SM), and knowledge (a non-theoretical construct). For each outcome measure, multiple regression analysis was used to examine the predictive value of each theoretical model individually. Following this 'theory level' analysis, a 'cross theory' analysis was conducted to investigate the combined predictive value of all significant individual constructs across theories.

**Results:**

All theories were tested, but only significant results are presented. When predicting behaviour, at the theory level, OLT explained 6% of the variance and, in a cross theory analysis, OLT 'evidence of habitual behaviour' also explained 6%. When predicting behavioural simulation, at the theory level, the proportion of variance explained was: TPB, 31%; SCT, 26%; II, 6%; OLT, 24%. GPs who reported having already decided to change their management to try to avoid the use of antibiotics made significantly fewer scenario-based decisions to prescribe. In the cross theory analysis, perceived behavioural control (TPB), evidence of habitual behaviour (OLT), CS-SRM cause (chance/bad luck), and intention entered the equation, together explaining 36% of the variance. When predicting intention, at the theory level, the proportion of variance explained was: TPB, 30%; SCT, 29%; CS-SRM 27%; OLT, 43%. GPs who reported that they had already decided to change their management to try to avoid the use of antibiotics had a significantly higher intention to manage URTIs without prescribing antibiotics. In the cross theory analysis, OLT evidence of habitual behaviour, TPB attitudes, risk perception, CS-SRM control by doctor, TPB perceived behavioural control and CS-SRM control by treatment entered the equation, together explaining 49% of the variance in intention.

**Conclusion:**

The study provides evidence that psychological models can be useful in understanding and predicting clinical behaviour. Taking a theory-based approach enables the creation of a replicable methodology for identifying factors that predict clinical behaviour. However, a number of conceptual and methodological challenges remain.

## Background

Clinical and health services research are continually producing new findings that may contribute to effective and efficient patient care. However, despite the considerable resources devoted to biomedical science, a consistent literature finding is that the transfer of research findings into practice is a slow and haphazard process. A range of studies conducted in the USA, Netherlands, Britain, Canada, and Australia have found that 30 to 40 percent of patients do not receive treatments of proven effectiveness, and, equally discouraging, up to 25 percent of patients receive unnecessary care care that is potentially harmful [1-3]. Upper respiratory tract infections (URTIs) comprising tonsillitis, pharyngitis, laryngitis, sinusitis, otitis media, and the common cold are frequent presenting conditions in primary care. Of these conditions, those that present with sore throat (tonsillitis, pharyngitis, laryngitis) are responsible for just over 50% of presentations, with otitis media adding another 25% [4]. These conditions are frequently treated with antibiotics, and rates of antibiotic prescribing have been increasing in the UK [5]. Interview studies [6,7] have shown that general practitioners (GPs) have a range of reasons why they prescribe antibiotics for sore throats. These include the feeling that patients 'want something done' or expect to receive a prescription; beliefs that, despite the evidence, antibiotics may help some patients and could do little harm; a concern to preserve and build relationships with patients; and workload factors. Other studies have found that GPs often feel uncomfortable about prescribing antibiotics [8], and that antibiotics are ten times more likely to be prescribed if the doctor perceives that a patient expects them [9].

However, 'the absolute benefits [of using antibiotics in the treatment of sore throat] are modest. Protecting sore throat sufferers against suppurative and non-suppurative complications in modern Western society can be achieved only by treating with antibiotics many who will derive no benefit.' [10,11]; similar considerations apply to otitis media [11]. Reducing antibiotic prescribing in the community by the 'prudent' use of antibiotics is seen as one way to slow the rise in antibiotic resistance [12,13] and appears safe, in children at least [14]. However, understanding of how best to achieve this is limited [15,16]. Ranji *et al*. reviewed 34 studies (reporting 41 trials) addressing treatment decisions (as opposed to drug choice decisions), most of which studied prescribing for acute respiratory infections [16]. All the interventions examined (clinician education, patient education, provision of delayed prescriptions, audit and feedback, clinician reminders and decision support systems, and financial and regulatory incentives) were effective at reducing prescribing (median absolute effect -8.9% (inter-quartile range -12.4% to -6.7%), but no individual strategy (or combination of strategies) was more effective at reducing prescribing. An apparent decline in prescribing in the UK is thought to be due to a decline in presentation to clinicians with no underlying decrease in prescribing to presenting cases [17].

Implementation research is the scientific study of methods to promote the uptake of research findings, and hence to reduce inappropriate care. It includes the study of influences on healthcare professionals' behaviour and interventions to enable them to use research findings more effectively. Over the past 15 to 20 years, a considerable body of implementation research has developed [18-20]. This research demonstrates that a wide range of empirically defined interventions can be effective. These span the range of strategies aimed at individuals (*e.g*., audit and feedback, reminders, outreach visiting), those aimed at organisation of care (*e.g*., case management, revision of roles, continuous quality improvement) through to financial and regulatory interventions. For example, Grimshaw *et al*. reviewed studies of interventions to promote the uptake of clinical guidelines and showed that all interventions were effective some of the time, with a median absolute effect size of approximately 9% [20]. However, all interventions had a range of effect sizes across the studies examining them, and the basis for choosing a particular intervention was usually not described. One consequence of this is when such studies are reviewed the lack of any common underlying framework means that they provide little detailed information to guide the choice, or optimise the components, of such complex interventions when they are introduced into routine care settings [21]. In order to minimise the number of costly 'real world' pragmatic implementation trials that need to be conducted, it is necessary to identify the 'active ingredients' in interventions that aim to change professional behaviour. Interventions could be effective for two reasons: they may contain components that effectively overcome the specific barriers encountered in relation to a particular practice; or they may contain components that are always effective in changing practice. Therefore, it is necessary to develop an understanding of the factors underlying clinical behaviour in order to identify what sorts of factors should be targeted in implementation interventions.

Theory has the potential to offer a generalisable underlying framework for studying behaviour, and explanations for clinical behaviour can be investigated using psychological theories that have been successful in predicting behaviour and behaviour change. A study by Walker *et al*. [22] used the theory of planned behaviour (TPB) [23] to investigate factors associated with prescribing antibiotics for patients with a sore throat amongst GPs. It showed that the impact of individual beliefs and perceptions on the strength of motivation to prescribe was high and included both evidence-based and non-evidence based factors. From this, clear predictions could be made about the factors that were likely to increase motivation to reduce prescribing. Using such an approach, with theoretical models to measure theory-based cognitions, offers the potential of a generalisable framework within which to consider factors influencing behaviour and the development of interventions to modify them. However this study, whilst predicting intention, did not predict behaviour.

The current study, one part of a larger project [24,25], aimed to investigate the use of a number of psychological theories (selected where there was good evidence of predictive value) to explore factors associated with the actual behaviour of GPs managing URTIs without antibiotics. Variables were drawn from the Theory of Planned Behaviour (TPB) [23], Social Cognitive Theory (SCT) [26,27], Operant Learning Theory (OLT) [28], Implementation Intentions (II) [29], Common Sense Self-Regulation Model (CS-SRM) [30], and an adaptation of the Stage Models (SM) [31,32]. These specific theories, which are described in detail elsewhere [24], were chosen because they vary in their emphasis. Some focus on motivation, proposing that motivation determines behaviour, and therefore the best predictors of behaviour are factors that predict or determine motivation (*e.g*., TPB). Some place more emphasis on factors that are necessary to predict behaviour in people who are already motivated to change (*e.g*., II). Others propose that individuals are at different stages in the progress toward behaviour change, and that predictors of behaviour may be different for individuals at different stages (*e.g*., Precaution Adoption Process). The specific models used in this study were chosen for three additional reasons. First, they have been rigorously evaluated with patients or with healthy individuals. Second, they allow us to examine the influence on clinical behaviour of perceived external factors, such as patient preferences as well as organisational barriers and facilitators. Third, they all explain behaviour in terms of variables that are amenable to change. The objective of this study was to identify those theoretical constructs that predicted clinical behaviour, behavioural simulation (as measured by the decisions made in response to five written clinical scenarios), and behavioural intention.

## Methods

This was a predictive study of the theory-based cognitions and clinical behaviours of general practitioners (GPs) from Scotland. Theory-based cognitions were collected by postal questionnaire survey. Behavioural data was collected from routinely available prescribing data, and planned analyses explored the predictive value of theory-based cognitions in explaining variance in the behavioural data.

### Design and participants

The design was a predictive study with predictor measures (theory-based cognitions) measured by a single postal questionnaire survey during the 12 month period to which the behavioural data related. Two interim outcome measures of stated intention and behavioural simulation were collected at the same time as the predictor measures. Behavioural data was collected from routinely available prescribing data.

Study participants were a random sample of GPs from Scotland selected from a list of all Scottish general practitioners by a statistician using a list of random sampling numbers.

### Predictor measures

Theoretically derived measures were developed following the protocols of Ajzen [23], Bandura [26,27], Connor and Sparks [33], Moss-Morris [34], and Francis *et al*. [35]. The cognition questions were developed from initial interviews with 14 GPs in Scotland who took part in a semi-structured interview of up to 40 minutes, as recommended for the theory of planned behaviour. The interviews use standard elicitation methods and covered the views and experiences about managing patients with an URTI. Responses were coded into belief domains (behavioural, normative, control) which were then used, in conjunction with the literature, to create the questions measuring constructs. Five knowledge questions were developed by the study team based on issues for which there was good evidence. Appendix 1 provides a summary of the predictor measures used in this study (see also [24]); the instrument is available as Additional File 1. Unless otherwise stated, all questions were rated on a seven-point scale from Strongly Disagree to Strongly Agree. We aimed to include at least three questions per psychological construct.

### Outcome measures

#### Behaviour

Our premise was that GPs who were more likely to manage URTIs without antibiotics would have lower antibiotic prescribing rates. Therefore, as a proxy for managing URTIs without antibiotics, the behavioural measure was each respondent's total number of antibiotic prescriptions. The raw data were adjusted in two ways. First, from the routine  prescribing data corresponding to chapter five (Infections) of the  British National Formulary (BNF) [36], although it was not possible to identify only those prescriptions that were given for uncomplicated URTIs, it was possible to exclude some antibiotics that would not be, or were very unlikely to have been, prescribed for URTIs. Some drugs were totally excluded (*e.g*., any anti-tuberculous drugs) and others were partly excluded on the basis of dose, dosage frequency and duration, and licensed indication (*e.g*., amoxicillin 3 g sachets, erythromycin in 90 day courses). Second, individual prescribing data was standardised by the number of patients the GP saw (our proxy measure of this was the number of half day sessions worked by each respondent).

Each prescription carries an identification code that is unique to the prescribing GP. However, it is possible that another clinician (*e.g*., a doctor in training) might use a respondent's prescriptions, resulting in an overestimate of the total number of prescriptions issued by that respondent. In order to allow us to make some estimate of this, all respondents were asked to estimate 'Over the last six months, how often have acute antibiotic prescriptions been written/printed by someone else (*e.g*., locum/trainee) using your cipher number?' with response options of Never, Sometimes, Frequently, Don't Know. The response to this question was used to conduct a sensitivity analysis.

#### Behavioural simulation

Key elements which might influence GPs' decisions to manage URTIs without antibiotics were identified from the literature, opinions of the clinical members of the research team, and the initial interviews with 14 GPs. From this, five clinical scenarios were constructed describing patients presenting in primary care with symptoms of an URTI (see Additional File 1). Respondents were asked to decide whether or not they would prescribe an antibiotic, and decisions in favour of prescribing an antibiotic were summed to create a total score out of a possible maximum of five.

#### Behavioural intention

Three questions assessed GP's intention to manage URTIs without antibiotics: When a patient presents with an URTI, I have in mind to prescribe an antibiotic, I intend to prescribe antibiotics for patients who present with an URTI as part of their management, I aim not to prescribe antibiotics for patients with URTI (rated on a seven-point scale from 'Strongly Disagree' to 'Strongly Agree'). Responses were summed (range 3 – 21) and scaled so that a low score equated with a low intention to prescribe antibiotics.

### Procedure

Participants were mailed an invitation pack (letter of invitation, questionnaire consisting of psychological and demographic measures, a form requesting consent to allow the research team to access the respondent's prescribing data, a study newsletter, and a reply paid envelope) by research staff between mid-April and mid-May 2004. Two postal reminders were sent to non-responders at two and four weeks. Behavioural data were collected over a one-year period, from approximately six months before to six months after the assessment of cognitions. The number of prescriptions for antibiotics issued between the beginning of November 2003 and the end of October 2004 were obtained from the Information and Statistics Division of Primary Care Information Group, Information Services, NHS National Services, Scotland.

### Sample size and statistical analysis

The target sample size of 200 was based on a recommendation by Green [37] to have a minimum of 162 subjects when undertaking multiple regression analysis with 14 predictor variables.

The overall analytic approach was to first check the internal consistency of the measures. Next, for each of the three outcome variables, we examined the relationship between predictor and outcome variables within the structure of each of the theories individually. Finally, for predictors that were statistically significant irrespective of whether or not they came from the same theory, we similarly examined the relationship between predictive and outcome variables. When comparing groups, independent t-tests were used as appropriate.

The internal consistency of the constructs measured with multiple questions was examined. Where necessary, questions were removed to achieve a Cronbach's alpha of 0.6 or greater. Where this was not possible the highest alpha was achieved. For two question constructs a correlation coefficient of 0.25 was used as a cut off. The relationship between predictive and outcome variables were examined using ANOVA for the Stage Model and correlation for other variables. Given that Implementation Intention (II) is theorized to act after intention and before behaviour, II is a post-intentional construct and therefore its prediction of intention was not explored.

For each of the three outcome measures, Pearson Correlation Coefficients between the individual constructs and the outcome measures were calculated, and then multiple regression analyses were used to examine the predictive value of each theoretical model. For the five 'perceived cause of illness' questions in the CS-SRM responses were dichotomized into scores of five to seven (indicating agreement that the cause in question was responsible for URTIs) versus anything else (indicating disagreement). These dichotomous variables then were entered as independent variables into the regression.

Finally, for predictors that were statistically significant, irrespective of whether or not they came from the same theory, we similarly examined the relationship between predictive and outcome variables. All constructs which predicted the outcome (p < 0.25 for a univariate relationship) were entered into a stepwise regression analysis to investigate the combined predictive value of significant constructs across all theories.

### Ethics approval

The study was approved by the UK South East Multi-Centre Research Ethics Committee.

## Results

**Figure 1:**
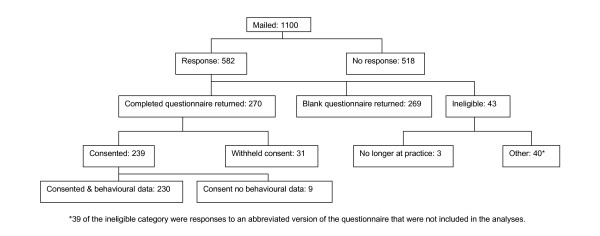
Response rates.

The postal questionnaire survey ran from mid-April to mid-May 2004. Of the 1,100 GPs approached, there were 230 (21%) who agreed to participate and for whom we could obtain prescribing data (Figure 1). Fifty-eight percent were male, they had been qualified for a mean (SD) of 21 (7.8) years, had a median (inter-quartile range (IQR)) list size of 6,900 (4,000 to 9,340), a median (IQR) of four (two to five) partners, and worked a median (IQR) of eight (six to nine) half-day sessions a week; 45 (18%) were trainers.

More respondents provided usable data on intention (261) than provided usable data on behavioural simulation (252). Both these figures were larger than the number of respondents who agreed to allow us to receive their behaviour data (227). Hence, the numbers included in analyses vary between the outcome measures.

### Relationship between the three outcome measures

The three outcome measures were significantly correlated with each other: for Behaviour and Behavioural Simulation, the Pearson r statistic was 0.17 (p = 0.013); similarly for Behaviour and Behavioural Intention it was 0.19 (p = 0.004); and for Behavioural Simulation and Behavioural Intention, it was 0.44 (p < 0.001).

### Predicting behaviour

The mean (SD) number of prescriptions issued was 57 (31) per 100 patients. The results of the correlation analyses are shown in Table 1. TPB attitudes, intention and perceived behavioural control, SCT risk perception, self-efficacy, action planning, OLT anticipated consequences, evidence of habitual behaviour and CS-SRM cause (chance/bad luck) significantly predicted the use of antibiotics to treat URTIs. For the Stage Model, the 167 GPs who endorsed that they had 'already changed my management of URTIs to try to avoid the use of antibiotics' issued a mean (SD) of 54 (30) prescriptions per 100 patients versus 66 (29) for the 52 GPs who endorsed any other response (mean difference (95%CI) = 11.8 (21.1 to 2.5), p = 0.014).

**Table 1:** Predicting behaviour by psychological theory: descriptive statistics, correlation and multiple regression analyses.

**Theoretical framework**	**Predictive Constructs**	**N**	**Alpha**	**Mean**	**(SD)**	**r**	**Beta**	**R2(adj)**	**df**	**F**
**Theory of Planned Behaviour (a)**	Attitude direct	3	0.54	8.7	(2.8)	0.136*				
	Attitude indirect	7	0.56	148.7	(35.7)	0.012				
	Subjective Norm	3	0.68	50.2	(18.8)	-0.103				
	
	Intention	3	0.68	6.5	(2.5)	0.193**	0.147*			
	PBC direct	4	0.70	17.0	(4.5)	-0.113	-0.013***			
	PBC power	7	0.86	24.4	(6.6)	0.171*	0.1**	0.033	3, 215	3.5*

**Social Cognitive Theory**	Risk perception	3	0.61	8.8	(2.8)	0.179**	0.183*			
	Outcome expectancies (self)	2	0.80	36.1	(15.0)	-0.052	-0.103			
	Outcome expectancies (behaviour)	7	0.56	18.5	(4.5)	-0.03	-0.133*			
	Self-efficacy	6	0.88	35.9	(11.1)	0.175**	0.155			
	Generalised self-efficacy	10	0.85	28.6	(3.6)	-0.005	0.045	0.049	5, 208	3.2**

**Implementation Intention**	Action Planning	-		2.9	(1.7)	0.169*	0.0169*	0.024	1, 220	6.4

**Operant Learning Theory**	Anticipated consequences	3	0.61	8.8	(2.8)	0.179**	0.087			
	Evidence of habitual behaviour	2	0.70	4.7	(2.1)	0.253***	0.218**	0.063	2, 216	8.3***

**Common Sense Self-regulation Model**	Identity of condition	2	0.57	7.4	(2.0)	-0.013	-0.019			
	Time (acute/chronic)	1		3.6	(1.2)	0.036	-0.056			
	Time (cyclical)	1		3.7	(1.3)	0.088	0.022			
	Control (by treatment)	2	0.27	5.6	(1.9)	0.097	0.181*			
	Control (by patient)	2	0.57	9.5	(2.2)	0.061	0.193*			
	Control (by doctor)	2	0.60	8.1	(2.4)	-0.01	-0.016			
	Cause: social contact	1		5.2	(1.0)	-0.121	-0.127			
	Cause: viral prevalence	1		5.5	(0.9)	-0.011	0.055			
	Cause: stress	1		3.7	(1.4)	-0.128	-0.105			
	Cause: air travel	1		4.7	(1.2)	-0.095	-0.018			
	Cause: chance/bad luck	1		4.3	(1.5)	-0.133*	-0.162*			
	Consequence	2	0.34	8.0	(3.0)	0.003	-0.047			
	Coherence	2	0.67	11.1	(1.8)	0.049	0.005			
	Emotional Response	4	0.63	9.6	(3.7)	0.102	0.111	0.028	14, 191	1.4

**Other**	Knowledge	5	0.00	2.9	(0.9)	-0.057	-0.057	0.000	1, 222	0.717

*p ≤ 0.05; ** p ≤ 0.01; ***p ≤ 0.001.

(a) Only intention and perceived behavioural control measures are entered into the regression equation as only these constructs are the proximal predictors of behaviour in this model.

Alpha = Cronbach's Alpha; r = Pearson product moment correlation coefficient; Beta = standardised regression coefficients; - = single question measure.

The results of the theory level analyses are shown in Table 1. The TPB explained 3% of the variance in behaviour, SCT explained 5%, and OLT explained 6%.

In the cross theory analysis, only evidence of habitual behaviour (OLT) was retained in the regression model, explaining 6% of the variance in the number of antibiotic prescription issued (Table 3).

#### Sensitivity analysis

Forty-five respondents for whom we also had behavioural data indicated that prescriptions had frequently been written/printed by someone else. Their mean (SD) number of prescriptions issued was 70 (31) per 100 patients compared to 55 (30) for respondents who answered anything else (p = 0.006). When the analyses were repeated excluding these respondents, there were no differences from the overall analysis.

### Predicting behavioural simulation

In response to the five clinical scenarios, the respondents indicated that they would prescribe for a mean (SD) of 1.6 (1.2) cases. The median number of prescriptions issued was one with a range of zero to five. From Table 2, the constructs which predicted behavioural simulation (*i.e*., what GPs said they would do in response to the specific clinical scenarios) were: TPB attitudes, perceived behavioural control and intention; SCT risk perception, outcome expectancies, and self-efficacy; action planning; OLT anticipated consequences and evidence of habitual behaviour; CS-SRM time (acute/chronic), control (by treatment), cause (chance/bad luck); and knowledge.

**Table 2:** Predicting behavioural simulation and intention by psychological theory: correlation and multiple regression analyses.

		**Behavioural simulation**					**Behavioural intention**				
**Theoretical framework**	**Predictive Constructs**	**r**	**Beta**	**R2(adj)**	**df**	**F**	**r**	**Beta**	**R2(adj)**	**df**	**F**

**Theory of Planned Behaviour**	Attitude direct	0.316***									
	Attitude indirect	0.212***									
	Subjective Norm	0.005									
	
	Intention		0.362***								
	PBC direct		-0.292***	0.267	2, 245	45.9***					
	
	Intention	0.440***	0.270***								
	PBC direct	-0.388***	-0.156***								
	PBC power	0.492***	0.278***	0.308	3, 244	37.6***					
	
	Attitude direct						0.469***	0.343***			
	Attitude indirect						0.228***	0.039			
	Subjective Norm						0.041	0.107			
	PBC direct						-0.264***	-0.019			
	PBC power						0.438***	0.288***	0.302	5, 239	22.1***

**Social Cognitive Theory**	Risk perception	0.350***	0.156*				0.461***	0.314***			
	Outcome expectancies (self)	0.191**	0.095				0.182**	0.125*			
	Outcome expectancies (behaviour)	0.265***	0.140*				0.217***	0.077			
	Self-efficacy	0.433***	0.355***				0.414***	0.268***			
	Generalised self-efficacy	-0.109	-0.025	0.259	5, 232	17.6***	-0.087	-0.016	0.289	5, 233	20.4***

**Implementation intention**	Action Planning	0.257***	0.257***	0.062	1, 249	17.6***					

**Operant Learning Theory**	Anticipated consequences	0.350***	0.196**				0.461***	0.245***			
	Evidence of habitual behaviour	0.457***	0.374***	0.240	2, 240	37.9***	0.621***	0.514***	0.426	2, 249	94.3***

**Common Sense Self-regulation Model**	Identity of condition	-0.063	-0.147				-0.043	-0.108			
	Time (acute/chronic)	0.148*	0.056				0.092	-0.014			
	Time (cyclical)	0.090	0.100				0.164**	0.060			
	Control (by treatment)	0.358***	0.388***				0.393***	0.476***			
	Control (by patient)	-0.028	0.130				0.001	0.160*			
	Control (by doctor)	0.102	0.110				0.188**	0.117			
	Cause: social contact	0.003	0.074				-0.042	0.020			
	Cause: viral prevalence	-0.051	-0.120				-0.089	-0.081**			
	Cause: stress	0.011	-0.036				-0.036	-0.094			
	Cause: air travel	0.049	0.023				-0.024	-0.011			
	Cause: chance/bad luck	0.140*	0.140*				-0.009	-0.008			
	Consequence	0.004	-0.111				0.173**	0.094			
	Coherence	-0.113	0.017				0.282***	0.155*			
	Emotional Response	0.070	0.071	0.160	16, 268	1.7	0.054	-0.017	0.272	14,221	7.3***

**Other**	Knowledge	-0.221***	-0.221***	0.045	1, 250	12.8***	-0.164**	-0.164**	0.023	1, 251	6.97**

*p ≤ 0.05; ** p ≤ 0.01; ***p ≤ 0.001.

r = Pearson product moment correlation coefficient; Beta = standardised regression coefficients.

The results of the theory level analyses are shown in Table 2. The TPB explained 31% of the variance in behavioural simulation, SCT explained 26%, II explained 6%, OLT explained 24%, and knowledge explained 4.5%. For the Stage Model, the 182 GPs who endorsed that they had 'already decided to change my management of URTIs to try to avoid the use of antibiotics' made a mean (SD) of 1.4 (1.1) decisions to prescribe versus 2.4 (1.3) for the 64 GPs who endorsed any other response (mean difference (95%CI) = -1.0 (1.2 to -0.7), p < 0.001).

In the cross theory analysis, perceived behavioural control (TPB), evidence of habitual behaviour (OLT), CS-SRM cause (chance/bad luck), and intention were retained in the regression model, together explaining 36% of the variance in the scenario score (Table 3).

**Table 3:** Results of the stepwise regression analyses which included all constructs which significantly predicted outcomes.

**Predictive Constructs**					
**Outcome: Prescribing antibiotics**	**Entered **	**Beta**	**Adj. R2**	**df**	**F**

TPB: Attitude Direct; Subjective Norm; PBC Power & PBC Power direct; Intention SCT: Risk Perception; Self-Efficacy Implementation Intentions: Action Planning Operant learning theory: anticipated consequences; Evidence of habitual behaviour CS-SRM: Cause social contact; stress; chance/bad luck	OLT Evidence of habitual behaviour	0.251***	0.059	1, 209	14.1***

**Outcome: Behavioural Simulation**					

TPB: Attitude Indirect & Direct; PBC Power & PBC Power direct; Intention SCT: Risk Perception; Outcome expectancy, Self-Efficacy; Generalised self-efficacy Implementation Intentions: Action Planning CS-SRM: Control treatment & doctor; Cause chance/bad luck; coherence Knowledge Operant learning theory: anticipated consequences; Evidence of Habitual Behaviour	TPB PBC Power	0.302***			
	OLT Evidence of habitual behaviour	0.237**			
	CS-SRM Cause chance/bad luck	0.154**			
	TPB Intention	0.178*	0.356	4, 220	31.92***

**Outcome: Behavioural Intention**					

TPB: Attitude Indirect & Direct; PBC Power & PBC Power direct SCT: Risk Perception; Outcome expectancy, Self-Efficacy; Generalised self-efficacy CS-SRM: Time cyclical; Control treatment & doctor; Consequence; Coherence Knowledge Operant learning theory: anticipated consequences; Evidence of Habitual Behaviour	OLT Evidence of habitual behaviour	0.410***			
	TPB attitudes direct	0.161**			
	SCT risk perception	0.149**			
	CS-SRM control doctor	0.142**			
	TPB PBC power	0.130*			
	CS-SRM control treatment	-0.108*	0.494	6, 224	38.36***

*p ≤ 0.05; **p ≤ 0.01; ***p ≤ 0.001.

PBC = perceived behavioural control; TPB = Theory of Planned Behaviour; SCT = Social Cognitive Theory; CS-SRM = Common Sense Self-Regulation Model.

### Predicting behavioural intention

With the range of possible scores for intention of 3 – 21, the mean (SD) intention score was 6.5 (2.5); the median intention score was 6 with a range of 3 to 14. The constructs which predicted behavioural intention were: TPB attitudes, perceived behavioural control; SCT risk perception, outcome expectancy, self-efficacy; OLT anticipated consequences, evidence of habitual behaviour; CS-SRM time (cyclical), control (by treatment and by doctor), consequences, coherence; and knowledge (Table 2).

The results of the theory level analyses are shown in Table 2. The TPB explained 30% of the variance in behavioural intention, SCT explained 29%, CS-SRM explained 27%, II explained 9%, OLT explained 43%, and knowledge and attitudes together explained 22%. For the Stage Model, the 188 GPs who endorsed that they had 'already decided to change my management of URTIs to try to avoid the use of antibiotics' had a mean (SD) intention score of 6 (2.3) versus 7.8 (2.6) for the 66 GPs who endorsed any other response (mean difference (95%CI) = -1.8 (2.5 to -1.3), p < 0.001).

In the cross theory analysis, OLT evidence of habitual behaviour, TPB attitudes, risk perception, CS-SRM control by doctor, TPB perceived behavioural control, and CS-SRM control by treatment were retained in the regression model, together explaining 49% of the variance in intention (Table 3).

## Discussion

We have successfully developed and applied psychological theory-based questionnaires that have been able to predict prescribing behaviour and two proxies for behaviour – behavioural simulation and intention.

### Overall interpretation

The management of URTI is a frequent behaviour, and our measure of self-reported habitual behaviour consistently predicted our outcome measures. Looking across our three outcome measures, there are also suggestions that issues of perceived control, risk perception, and attitudes may also be important.

The theories individually explained a significant proportion of the variance in our dependent variables, but the aggregated analysis suggested that they were measuring similar phenomena within their own individual structures. Our measure of habit was consistently identified as important, a finding that was supported by the result of the Stage Model analysis (albeit analysed as only two stages) which suggested that many GPs had already decided to prescribe fewer antibiotics. Because encouraging the implementation of any evidence-based practice commonly entails various methods of increasing knowledge, knowledge was included as a predictive construct in this study. The knowledge measure included questions about both how and why antibiotics might be used in the management of URTIs. The number of questions answered correctly was not related to the number of antibiotic prescriptions issued but was related to the behavioural simulation and intention scores. However, knowledge did not enter into any of the three stepwise regressions, indicating that other constructs are consistently more important and suggesting that behaviour change strategies aimed at changing knowledge alone are unlikely to be successful in this clinical area.

The stepwise regression analyses revealed that the main construct driving GPs' management of URTI was habit with additional influence from control, attitudes, and risk perception. Taken together, the results suggest that GPs have considered this frequently performed behaviour and operate in a predominantly habitual manner backed up by beliefs that support their habit.

This is a correlational study, so the causative aspects of the theories remain untested in this population; but it is promising for the utility of applying psychological theory to changing clinical behaviour that the constructs are acting as the theories expect. These results suggest that an intervention that specifically targets these elements should have the greatest likelihood of success in influencing the implementation of this evidence-based practice.

We used a range of theories and models in both this and another component [25] of our larger study [24]. However, across the two studies of different behaviours (URTIs, taking dental radiographs) and different clinicians (GPs, dentists), different constructs predicted different proportions of the variance in the intention and behaviour. This raises the question of what would be an optimum core set of measures if the aim was to cover most behaviours and clinical groups. Given our current limited understanding, this would have to be the subject of both studies replicating this one and further work examining different combinations of theories and models.

### Strengths and weaknesses

Operationalising the constructs with theoretical purity was a challenge. The preliminary study revealed that it was difficult to ask clinicians about their control over prescribing antibiotics because they believed that, even if they felt there were barriers to performing the behaviour, ultimately they had total control because they wrote the prescription. In the final questionnaire, this meant some questions had to be worded in terms of not doing the behaviour. There was some concern that not prescribing antibiotics may represent a range of alternative behaviours rather than being just a negative reflection of prescribing antibiotics.

A number of the models (OLT, II, CS-SRM) have not previously been operationalised in this way. OLT and II have usually being used as intervention methods to change behaviour. However, they both have been able to predict behavioural simulation, and OLT predicted intention and behaviour. The CS-SRM did not predict significant variance in behaviour or behavioural simulation, but the model did explain 27% of the variance in intention, a similar proportion to both TPB and SCM. The model has previously been used mainly to refer to an individual's perceptions of their clinical condition; we used it to measure a clinician's perception of the condition in general. We had difficulty operationalising this model, and further work is needed to explore how best the model can be applied to clinician's behaviour in respect of their patients.

One of the main strengths of this study is that the primary outcome was behaviour. The inclusion of the self-reported secondary outcomes of behavioural intention and simulation made it possible to examine the relationship between these three measures. This is important because behaviour is usually more difficult (and expensive) to measure than either of these proxy measures. By virtue of their significant correlation, the results suggest that self-reported measures have the potential to proxy behavioural data when testing an intervention prior to implementation in a service-level trial. However, although the two proxy measures (intention and simulation) were moderately correlated, the correlation between either and behaviour was weak. It is possible that the proxy measures are poor predictors of behaviour, though it is important to remember that the models we have used are focussing on modifiable behaviour. This cannot be quantified in our predictive study design but will only ever be a small proportion of behaviour. However, it is also important to consider the validity of our behaviour measure.

There is a stepwise decrease in the proportion of variance explained as we move from explaining intention to behavioural simulation to behaviour, with the models that we used explaining up to 49%, 36% and 6% of the variance respectively (Table 3). In a meta analysis of TPB studies in the general population, Armitage and Conner [38] reported TPB explaining 31% of the variance in self-reported behaviour and 20% in observed behaviour. Our data explaining up to 34% of the variance in behavioural simulation is very similar to Armitage and Connor's figure for self-reported behaviour, while our explaining up to 6% of the variance in behaviour is lower than their figure of 20%. In a parallel study using identical methods, we have been able to explain 16% of the variance in general dental practitioners' use of dental radiographs [25]. This suggests that our operationalisation of the models was good, but that either the models do not work for this behaviour in GPs or there are problems with our measure of behaviour, or both. A systematic review [39] found only 10 studies exploring the relationship between intention and behaviour in healthcare professionals, but these reported explaining a similar proportion of the variance in observed behaviour to the studies in Armitage and Connor's review [38]. This suggests that the problem is with our measure of behaviour.

There could be two problems – prescribing data may not be a good proxy for the behaviour as we asked about it in our questionnaires, or there may be biases within prescribing data. We have already identified the potential problem with using antibiotic prescription as a proxy for the management of patients with URTIs without prescribing antibiotics. Not only may 'prescribing' not be the reverse of 'not prescribing', 'not prescribing' may represent a number of alternate behaviours. Prescribing data was chosen because it was available from routine data sources, and was therefore inexpensive to collect. Antibiotic prescribing was chosen because it was more likely that a prescription for an acute illness (as opposed to a chronic illness managed through a repeat prescribing system) would be attributed to the GP who issued it. Scotland was chosen because the most commonly used computer system was likely to ascribe an antibiotic prescription to the issuing doctor. Despite this, we know that there are errors in the attribution of prescriptions to doctors, with 45 respondents reporting that prescriptions had frequently been written in their name by someone else. Finally, our standardisation by the number of patients registered with the GP assumes that each doctor has the same presentation rate. We sought to minimise variation in this by measuring over a 12-month period, but it is possible that this was still a problem. In future studies of this kind it will be important to invest more in the measurement of the behavioural data.

Our final response rate was not high compared to what would be expected for a postal questionnaire survey. Cummings *et al*. reported that up to 1995, response rates of surveys of healthcare professionals remained constant at approximately 60% [40]. Our previous study using similar questionnaires to investigate antibiotic prescribing had a response rate of 68% [22]. Kaner *et al*. reported doctors describing day to day work pressures and lack of perceived salience as reasons for not completing questionnaires [41]. Since these three studies, day-to-day work pressures in UK NHS primary care have continued to rise, and our operationalisation of multiple models resulted in a long questionnaire asking seemingly repetitive questions. Additionally, our request to access behavioural data deterred 31 respondents who returned a completed questionnaire; it may have deterred a larger group from even completing a questionnaire.

Although we cannot make direct comparisons, our respondents appear well-matched with the overall population of Scottish GPs on gender, age and prescribing rates but came from larger practices and were more likely to be trainers. From publicly available data (see ISD Scotland [42]) for 2003 and 2004, demographic data for all Scottish GPs were: 55% male, mean age 44.7 years (assuming qualification at age 23, this gives 21.7 years qualified), average practice size of 5,089, and 10% were trainers. Mean national rates of antibiotic prescribing in 2004 (having made, where possible, similar exclusions to those made in this study) was 65 prescriptions per 100 patients. Therefore, while we should be cautious about generalising from our respondents to the population of Scottish GPs, this is less of an issue at this exploratory stage of using these methods. Our aim was not to generate data that was representative but to receive our pre-specified number of responses from a population who had a range of behaviour, reported a range of behavioural simulation and intention, and who reported a range of cognitions. The study achieved this aim.

## Conclusion

This study provides evidence that psychological models can be useful in understanding and predicting clinical behaviour. Taking a theory-based approach enables the creation of a replicable methodology for identifying factors which predict clinical behaviour. However, there remain conceptual challenges in operationalising a number of the models and a range of methodological challenges in terms of instrument development and measurement of behaviour that have to be surmounted before these methods could be regarded as routine.

## Competing interests

Martin Eccles is Co-Editor in Chief of *Implementation Science*; Jeremy Grimshaw is a member of the editorial board of *Implementation Science*. All editorial decisions on this article were made by Co-Editor in Chief Brian Mittman.

## Authors' contributions

AW, ME, JG, MJ, NP conceived the study. MJ, LS, GM, RT, DB and ME contributed to the daily running of the study. MJ and NS oversaw the analysis which was conducted by GM. All authors commented on sequential drafts of the paper and agreed the final draft.

## Appendix 1

Table 4 Contains a summary of the predictor measures.

**Table 4:** 

**Constructs (number of questions)**	**Example Question(s)**
**Theory of Planned Behaviour [23]**	
Behavioural intention (3)	I intend to prescribe antibiotics for patients who present with an URTI as part of their management
Attitude: Direct (3); Indirect^a^ (8 behavioural beliefs (bb) multiplied by 8 outcome evaluations (oe). The score was the mean of the summed multiplicatives.)	Direct: In general: The possible harms of antibiotics to patients with an URTI outweighs their benefits; Indirect: In general, prescribing an antibiotic for a patient with an URTI would reassure them (bb) × reassuring the patient is (oe: un/important)
Subjective Norm^b^: Indirect (5 normative beliefs (nb) multiplied by 5 motivation to comply (mtc) questions. The score was the mean of the summed multiplicatives).	When managing URTIs, I feel under pressure not to prescribe an antibiotic: from published literature (nb) × How motivated are you to do what the published literature states that you should (mtc: very much/not at all)
Perceived Behavioural Control: Direct (4); Indirect/power (7)^c^	Direct: Whether I manage an URTI without prescribing an antibiotic is entirely up to me Indirect: I find it difficult to manage patients presenting with an URTI without prescribing an antibiotic who: Expect me to prescribe an antibiotic
**Social Cognitive Theory [26]**	
Risk Perception (3)	It is highly likely that patients with an URTI will be worse off if I do not prescribe an antibiotic.
Outcome Expectancies Self (2 × 2), Behaviour (8 × 8). The score was the mean of the summed multiplicatives.	Self: If I do not prescribe an antibiotic for a patient with an URTI, then I will think of myself as a competent GP × Thinking of myself as a competent GP is (Un/Important). Behaviour: See Attitude (Theory of Planned Behaviour)
Self-Efficacy: General: Generalized Self-Efficacy Scale [43] (10: 4 point scale, not at all true/exactly true); Specific (7)	General: I can always manage to solve difficult problems if I try hard enough Specific: How confident are you in your ability to manage patients with URTIs symptomatically
**Implementation Intention [29]**	
Action planning (1)	Currently, my standard method of managing patients with an URTI does not include prescribing an antibiotic
**Operant Learning Theory [28]; BF Skinner Foundation **[44]	
Anticipated consequences (3)	If I do not routinely prescribe antibiotics for URTIs then, on balance, my life as a GP will be easier in the long run
Evidence of habit (2)	When I see patients with URTIs, I automatically consider managing them without an antibiotic
Experienced (rewarding and punishing) consequences (4: more likely to prescribe (score = 1); less likely (score = -1); unchanged/not sure/never occurred (score = 0)). Scores were summed.	Think about the last time you prescribed an antibiotic for a patient with an URTI and felt pleased/sorry: Think about the last time you decided not to prescribe an antibiotic for a patient with an URTI and felt pleased/sorry that you had not done so':
**Common Sense Self-Regulation Model^d^ [30]**	
Perceived identity (3)	URTIs as seen in general practice generally have symptoms of an intense nature
Perceived cause (5)	Getting a URTI is determined by stress
Perceived controllability (patient, doctor, treatment) (6)	What the patient does can determine whether an URTI gets better or worse
Perceived duration (acute/chronic; cyclical) (3)	URTIs as seen in general practice are very unpredictable
Perceived consequences (3)	An URTI does not have much effect on a patient's life
Coherence (2)	I have a clear picture or understanding of URTIs
Emotional response (4)	Seeing patients with an URTI does not worry me
**Stage Model [31,32]**	
Current stage of change. A single statement is ticked to indicate the behavioural stage	Unmotivated (2): I have not/it has been a while since I have thought about changing my management of URTIs to try to avoid the use of antibiotics. Motivated (2): I have decided that I will/will not change my management of URTIs to try to avoid the use of antibiotics. Action (1): I have already changed my management of URTIs to try to avoid the use of antibiotics.
**Other Measures**	
Knowledge (5) (True/False/Not Sure) Demographics	The presence of pus on the tonsils suggests a bacterial infection post code, gender, time qualified, number of other doctors in practice, trainer status, hours per week, list size

^a^All indirect measures consist of specific belief questions identified in the preliminary study as salient to the management of upper respiratory tract infections.

^b^These individuals and groups were identified in the preliminary study as influential in the management of upper respiratory tract infections

^c^An indirect measure of perceived behavioural control usually would be the sum of a set of multiplicatives (control beliefs × power of each belief to inhibit/enhance behaviour). However, the preliminary study demonstrated that it proved problematic to ask clinicians meaningful questions which used the word 'control' as clinicians tended to describe themselves as having complete control over the final decision to perform the behaviour. Support for measuring perceived behavioural control using only questions as to the ease or difficulty of performing the outcome behaviour was derived from a metanalysis which suggested that perceived ease/difficulty questions were sensitive predictors of behavioural intention and behaviour [45].

^d^Illness representation measures were derived from the Revised Illness Perception Questionnaire [34]

## Supplementary Material

Additional file 1 Questionnaire Click here for file
