# Supplementary material for: Applying psychological theories to evidence-based clinical practice: Identifying factors predictive of managing upper respiratory tract infections without antibiotics
Source: Implement Sci. 2007 Aug 3;2:26. doi: 10.1186/1748-5908-2-26 (PMC2042498; doi:10.1186/1748-5908-2-26)
Supplement: Additional file 1 — Questionnaire [file 1748-5908-2-26-S1.pdf]

PR1ME

# MANAGEMENT OF PATIENTS WITH AN UPPER RESPIRATORY TRACT INFECTION IN PRIMARY CARE

HEALTH SERVICES RESEARCH UNIT

UNIVERSITY OF NEWCASTLE

DENTAL HEALTH SERVICES RESEARCH UNIT

MRC Medical Research Council

## INTRODUCTION

**Thank you for participating in this project. This questionnaire refers to factors influencing management of patients presenting with an upper respiratory tract infection (URTI) in general practice. For the purposes of this questionnaire, URTI includes sore throat, nasal discharge and coughs. Some of the questions seem to be very alike. They are different and it is important to answer them all. Most questions are answered by circling one number; a few require more time to answer. Try not to take to long over each response we would like to know your immediate views and experiences. Your answers are completely confidential**

### Section 1: PRESCRIBING ANTIBIOTICS

**1.** a) **From memory, approximately how many of the last 10 patients who presented with an URTI for the first time, did you manage *without* prescribing an antibiotic:**

0 1 2 3 4 5 6 7 8 9 10

b) **From memory, approximately how many of the last 10 patients who re-presented with the same URTI episode, did you manage *without* prescribing an antibiotic:**

0 1 2 3 4 5 6 7 8 9 10

**2. When managing URTIs, I feel under pressure to prescribe an antibiotic:**

| | *Strongly disagree* | | | | | | *Strongly agree* |
|---|---|---|---|---|---|---|---|
| a) from patients | 1 | 2 | 3 | 4 | 5 | 6 | 7 |
| b) from secondary care colleagues | 1 | 2 | 3 | 4 | 5 | 6 | 7 |

**3. When managing URTIs, I feel under pressure not to prescribe an antibiotic:**

| | *Strongly disagree* | | | | | | *Strongly agree* |
|---|---|---|---|---|---|---|---|
| a) from colleagues within the practice | 1 | 2 | 3 | 4 | 5 | 6 | 7 |
| b) from feedback provided by SPA | 1 | 2 | 3 | 4 | 5 | 6 | 7 |
| c) from published literature | 1 | 2 | 3 | 4 | 5 | 6 | 7 |

| | | | | | | | |
|---|---|---|---|---|---|---|---|
| **4. If I do not prescribe an antibiotic for a patient with an URTI, then I will think of myself as a caring GP** | 1 | 2 | 3 | 4 | 5 | 6 | 7 |
| **5. If I do not prescribe an antibiotic for a patient with an URTI, then I will think of myself as a competent GP** | 1 | 2 | 3 | 4 | 5 | 6 | 7 |
| **6. It is highly likely that patients with an URTI will be worse off if I do not prescribe an antibiotic** | 1 | 2 | 3 | 4 | 5 | 6 | 7 |

**7. In general, <u>prescribing</u> an antibiotic for a patient with an URTI would:**

| | | *Strongly disagree* | | | | | | *Strongly agree* |
|---|---|---|---|---|---|---|---|---|
| a) | Reassure them | 1 | 2 | 3 | 4 | 5 | 6 | 7 |
| b) | Alleviate their symptoms | 1 | 2 | 3 | 4 | 5 | 6 | 7 |
| c) | Increase their satisfaction with my care | 1 | 2 | 3 | 4 | 5 | 6 | 7 |
| d) | Make them more likely to expect an antibiotic for a URTI in the future | 1 | 2 | 3 | 4 | 5 | 6 | 7 |
| e) | Mean that the patient will not consult for the same URTI episode | 1 | 2 | 3 | 4 | 5 | 6 | 7 |
| f) | Reduce the time taken for their URTI to resolve | 1 | 2 | 3 | 4 | 5 | 6 | 7 |
| g) | Reduce the length of the consultation | 1 | 2 | 3 | 4 | 5 | 6 | 7 |
| h) | Increase the likelihood of antibiotic resistance in the community | 1 | 2 | 3 | 4 | 5 | 6 | 7 |

**8. If I do not routinely prescribe antibiotics for URTIs then:**

| | | *Strongly disagree* | | | | | | *Strongly agree* |
|---|---|---|---|---|---|---|---|---|
| a) | On balance, my life as a GP will be easier in the long run | 1 | 2 | 3 | 4 | 5 | 6 | 7 |
| b) | On balance, the consequences for me as a GP (e.g. stress, time, future consultations etc.) will be worse in the long run | 1 | 2 | 3 | 4 | 5 | 6 | 7 |

**9. How confident are you in your ability:**

| | | *Not at all confident* | | | | | | *Extremely confident* |
|---|---|---|---|---|---|---|---|---|
| a) | To treat URTIs without prescribing an antibiotic | 1 | 2 | 3 | 4 | 5 | 6 | 7 |
| b) | To end a consultation for a patient with an URTI without prescribing an antibiotic | 1 | 2 | 3 | 4 | 5 | 6 | 7 |
| c) | Not to prescribe an antibiotic if the patient's URTI symptoms are distressing them | 1 | 2 | 3 | 4 | 5 | 6 | 7 |
| d) | To manage patients with URTIs symptomatically | 1 | 2 | 3 | 4 | 5 | 6 | 7 |

**10.**

| | | *Strongly disagree* | | | | | | *Strongly agree* |
|---|---|---|---|---|---|---|---|---|
| a) | When I see patients with URTIs, I automatically consider managing them without an antibiotic | 1 | 2 | 3 | 4 | 5 | 6 | 7 |
| b) | It is my usual practice not to prescribe antibiotics for patients with URTIs | 1 | 2 | 3 | 4 | 5 | 6 | 7 |
| c) | I aim *not* to prescribe antibiotics for patients with URTI | 1 | 2 | 3 | 4 | 5 | 6 | 7 |
| d) | In my experience, antibiotics are more likely to successfully treat URTIs than symptomatic treatment alone | 1 | 2 | 3 | 4 | 5 | 6 | 7 |

**11. Given 10 patients presenting for the <u>first time</u> with the an URTI, how many patients would you expect to manage *without* prescribing an antibiotic?**

0 1 2 3 4 5 6 7 8 9 10

**12.** **Given 10 patients presenting for the second time with the same episode of an URTI, how many patients would you expect to manage *without* prescribing an antibiotic?**

0 1 2 3 4 5 6 7 8 9 10

**13.** **Which of these sentences *most* characterises you at the moment (Please tick *only one* of the 5 boxes)**

- ☐1 I have not thought about changing my management of URTIs to try to avoid the use of antibiotics
- ☐2 It has been a while since I have thought about changing my management of URTIs to try to avoid the use of antibiotics
- ☐3 I have decided that I *will not change* my management of URTIs to try to avoid the use of antibiotics
- ☐4 I have decided that I *will change* my management of URTIs to try to avoid the use of antibiotics
- ☐5 I have *already changed* my management of URTIs to try to avoid the use of antibiotics

**14.** a) Think about the last time you prescribed an antibiotic for a patient with an URTI and felt pleased that you had done so. Do you think the result of this episode has made you:

**More likely to prescribe** ☐ **Less likely to prescribe** ☐ **Unchanged** ☐ **Not sure** ☐ **Never occurred** ☐

b) Think about the last time you prescribed an antibiotic for a patient with an URTI and felt sorry that you had done so. Do you think the result of this episode has made you:

**More likely to prescribe** ☐ **Less likely to prescribe** ☐ **Unchanged** ☐ **Not sure** ☐ **Never occurred** ☐

c) Think about the last time you decided not to prescribe an antibiotic for a patient with an URTI and felt sorry that you had not done so. Do you think the result of this episode has made you:

**More likely to prescribe** ☐ **Less likely to prescribe** ☐ **Unchanged** ☐ **Not sure** ☐ **Never occurred** ☐

d) Think about the last time you decided not to prescribe an antibiotic for a patient with an URTI and felt pleased that you had not done so. Do you think the result of this episode made you::

**More likely to prescribe** ☐ **Less likely to prescribe** ☐ **Unchanged** ☐ **Not sure** ☐ **Never occurred** ☐

## SECTION 2: MANAGING PATIENTS PRESENTING WITH AN UPPER RESPIRATORY TRACT INFECTION

**1.** **I find it difficult to manage patients presenting with an URTI without prescribing an antibiotic who:**

| | *Strongly disagree* | | | | | | *Strongly agree* |
|---|---|---|---|---|---|---|---|
| a) Have already tried to self medicate for an URTI | 1 | 2 | 3 | 4 | 5 | 6 | 7 |
| b) Expect me to prescribe an antibiotic | 1 | 2 | 3 | 4 | 5 | 6 | 7 |
| c) Have a past history of an Chronic Obstructive Airways Disease | 1 | 2 | 3 | 4 | 5 | 6 | 7 |

**1. Generally I find it difficult:**

| | | *Strongly disagree* | | | | *Strongly agree* | | |
|---|---|---|---|---|---|---|---|---|
| a) | To treat patients with URTIs without prescribing an antibiotic | 1 | 2 | 3 | 4 | 5 | 6 | 7 |
| b) | To end a consultation with a patient with an URTI without prescribing an antibiotic | 1 | 2 | 3 | 4 | 5 | 6 | 7 |
| c) | Not to prescribe an antibiotic if the patient's URTI symptoms are distressing them | 1 | 2 | 3 | 4 | 5 | 6 | 7 |
| d) | To manage patients with an URTI symptomatically | 1 | 2 | 3 | 4 | 5 | 6 | 7 |

**3.**

| | | *Strongly disagree* | | | | *Strongly agree* | | |
|---|---|---|---|---|---|---|---|---|
| a) | I would like to avoid prescribing antibiotics for URTIs, but I don't really know if I can | 1 | 2 | 3 | 4 | 5 | 6 | 7 |
| b) | Whether I manage an URTI without prescribing an antibiotic is entirely up to me | 1 | 2 | 3 | 4 | 5 | 6 | 7 |
| c) | I am confident that I can avoid prescribing an antibiotic for an URTI whenever I want to | 1 | 2 | 3 | 4 | 5 | 6 | 7 |
| d) | I can overcome all obstacles, whatever they may be, in managing an URTI without a prescribing an antibiotic | 1 | 2 | 3 | 4 | 5 | 6 | 7 |

**4. In general:**

| | | *Strongly disagree* | | | | *Strongly agree* | | |
|---|---|---|---|---|---|---|---|---|
| a) | The possible harms of antibiotics to patients with an URTI outweighs their benefits | 1 | 2 | 3 | 4 | 5 | 6 | 7 |
| b) | Not prescribing antibiotics for patients with URTIs is more often bad practice than good | 1 | 2 | 3 | 4 | 5 | 6 | 7 |
| c) | Not prescribing antibiotics for patients with URTIs is more often unsatisfying than satisfying | 1 | 2 | 3 | 4 | 5 | 6 | 7 |

**5. In general:**

| | | *Unimportant* | | | | | | *Important* |
|---|---|---|---|---|---|---|---|---|
| a) | Reassuring patients is | 1 | 2 | 3 | 4 | 5 | 6 | 7 |
| b) | Alleviating patient symptoms is | 1 | 2 | 3 | 4 | 5 | 6 | 7 |
| c) | Increasing patient satisfaction with my care is | 1 | 2 | 3 | 4 | 5 | 6 | 7 |
| d) | Reducing their expectation of an antibiotic for a URTI in the future is | 1 | 2 | 3 | 4 | 5 | 6 | 7 |
| e) | Reducing the likelihood that the patient will consult again for the same URTI episode is | 1 | 2 | 3 | 4 | 5 | 6 | 7 |
| f) | Reducing the time taken for a patient's URTI to resolve is | 1 | 2 | 3 | 4 | 5 | 6 | 7 |
| g) | Reducing the length of consultations for URTIs | 1 | 2 | 3 | 4 | 5 | 6 | 7 |
| h) | Reducing antibiotic resistance is | 1 | 2 | 3 | 4 | 5 | 6 | 7 |

| | | | | | | | |
|---|---|---|---|---|---|---|---|
| **6. Thinking of myself as a caring GP is** | 1 | 2 | 3 | 4 | 5 | 6 | 7 |
| **7. Thinking of myself as a competent GP is** | 1 | 2 | 3 | 4 | 5 | 6 | 7 |

8. **How motivated are you to do what:**

| | | *Not at all* | | | | | | *Very much* |
|---|---|---|---|---|---|---|---|---|
| a) | Patients think you should | 1 | 2 | 3 | 4 | 5 | 6 | 7 |
| b) | Secondary care colleagues think you should | 1 | 2 | 3 | 4 | 5 | 6 | 7 |
| c) | Colleagues in primary care think you should | 1 | 2 | 3 | 4 | 5 | 6 | 7 |
| d) | SPA feedback states that you should | 1 | 2 | 3 | 4 | 5 | 6 | 7 |
| e) | The published literature states that you should | 1 | 2 | 3 | 4 | 5 | 6 | 7 |

9. **Without an antibiotic, how confident are you in your ability to manage patients with URTIs who:**

| | | *Not at all confident* | | | | | | *Extremely confident* |
|---|---|---|---|---|---|---|---|---|
| a) | Have already tried to self medicate for an URTI | 1 | 2 | 3 | 4 | 5 | 6 | 7 |
| b) | Expect you to prescribe an antibiotic | 1 | 2 | 3 | 4 | 5 | 6 | 7 |
| c) | Have a past history of Chronic obstructive airways disease | 1 | 2 | 3 | 4 | 5 | 6 | 7 |

| | | *Strongly disagree* | | | | | | *Strongly agree* |
|---|---|---|---|---|---|---|---|---|
| 10. | **When a patient presents with an URTI, I have in mind to prescribe an antibiotic** | 1 | 2 | 3 | 4 | 5 | 6 | 7 |
| 11 | **I intend to prescribe antibiotics for patients who present with an URTI as part of their management** | 1 | 2 | 3 | 4 | 5 | 6 | 7 |
| 12 | **Currently, my standard method of managing patients with an URTI does not include prescribing an antibiotic** | 1 | 2 | 3 | 4 | 5 | 6 | 7 |

13. a) **Do you have a clear idea how you would want to manage patients with an URTI?**

Yes ☐ No ☐ Unsure ☐

b) **If Yes, please describe it briefly**

________________________________________

________________________________________

________________________________________

________________________________________

________________________________________

## SECTION 3: UPPER RESPIRATORY TRACT INFECTIONS

**1. URTI as seen in general practice:**

| | | *Strongly disagree* | | | | | | *Strongly agree* |
|---|---|---|---|---|---|---|---|---|
| a) | Generally have symptoms of an intense nature | 1 | 2 | 3 | 4 | 5 | 6 | 7 |
| b) | Have many symptoms | 1 | 2 | 3 | 4 | 5 | 6 | 7 |
| c) | Should concern doctors | 1 | 2 | 3 | 4 | 5 | 6 | 7 |
| d) | Will pass quickly | 1 | 2 | 3 | 4 | 5 | 6 | 7 |
| e) | Are very unpredictable | 1 | 2 | 3 | 4 | 5 | 6 | 7 |
| f) | Change a great deal from day to day | | | | | | | |

**2. Getting a URTI is determined by:**

| | | *Strongly disagree* | | | | | | *Strongly agree* |
|---|---|---|---|---|---|---|---|---|
| a) | Social contact | 1 | 2 | 3 | 4 | 5 | 6 | 7 |
| b) | A high prevalence of viruses in the community | 1 | 2 | 3 | 4 | 5 | 6 | 7 |
| c) | Stress | 1 | 2 | 3 | 4 | 5 | 6 | 7 |
| d) | Air travel | 1 | 2 | 3 | 4 | 5 | 6 | 7 |
| e) | Chance or bad luck | 1 | 2 | 3 | 4 | 5 | 6 | 7 |

| | | *Strongly disagree* | | | | | | *Strongly agree* |
|---|---|---|---|---|---|---|---|---|
| **3.** | **There is a lot which the patient can do to control the symptoms of an URTI** | 1 | 2 | 3 | 4 | 5 | 6 | 7 |
| **4.** | **An URTI can be controlled by symptomatic treatment** | 1 | 2 | 3 | 4 | 5 | 6 | 7 |
| **5.** | **What the patient does can determine whether an URTI gets better or worse** | 1 | 2 | 3 | 4 | 5 | 6 | 7 |
| **6.** | **Nothing I do will influence the patient's URTI** | 1 | 2 | 3 | 4 | 5 | 6 | 7 |
| **7.** | **What I do can determine whether the patient's URTI gets better or worse** | 1 | 2 | 3 | 4 | 5 | 6 | 7 |
| **8.** | **In general, an URTI is a serious condition** | 1 | 2 | 3 | 4 | 5 | 6 | 7 |
| **9.** | **An URTI does not have much effect on the patient's life** | 1 | 2 | 3 | 4 | 5 | 6 | 7 |
| **10.** | **An URTI has serious financial consequences** | 1 | 2 | 3 | 4 | 5 | 6 | 7 |
| **11.** | **I have a clear picture or understanding of URTIs** | 1 | 2 | 3 | 4 | 5 | 6 | 7 |
| **12.** | **The symptoms of an URTI are puzzling to me** | 1 | 2 | 3 | 4 | 5 | 6 | 7 |
| **13.** | **I get depressed when I think about patients suffering with an URTI** | 1 | 2 | 3 | 4 | 5 | 6 | 7 |
| **14.** | **Seeing patients with an URTI does not worry me** | 1 | 2 | 3 | 4 | 5 | 6 | 7 |
| **15.** | **Seeing patients with an URTI makes me feel angry** | 1 | 2 | 3 | 4 | 5 | 6 | 7 |
| **16.** | **Seeing patients with an URTI can affect me emotionally (e.g. can make me feel helpless, anxious, or distressed)** | 1 | 2 | 3 | 4 | 5 | 6 | 7 |

## SECTION 4: MANAGING PATIENTS WITH AN UPPER RESPIRATORY TRACT INFECTIONS

| | | | *True* | *False* | *Not sure* |
|---|---|---|---|---|---|
| **1.** | a) | Antibiotics shorten the course of URTIs by 12-24 hours | 1 | 2 | 3 |
| | b) | The presence of pus on the tonsils suggests a bacterial infection | 1 | 2 | 3 |
| | c) | Symptomatic treatment is appropriate for the majority of people with URTIs | 1 | 2 | 3 |
| | d) | Antibiotics improve the symptoms of URTIs | 1 | 2 | 3 |
| | e) | If an antibiotic is used for an URTI, the antibiotic of choice (in an adult with no drug allergies) is | | | |

Penicillin ☐ Amoxicillin ☐ Flucloxacillin ☐ Cephalexin ☐ Othe ☐

The following scenarios include elements that may influence your decision to prescribe an antibiotic for a patient with an URTI. We appreciate the skills you may normally draw on during an actual consultation cannot be a factor in your decision. Please try and complete based on the information presented.

**2**. The first patient is a 69-year-old man. This is his first visit to the surgery. He does not often visit the surgery, and lives alone. He has had a sore throat and a chesty cough. He tells you he has COPD. He tells you that he is not particularly worried about his condition, but he wants to be sure that it's not important, as things don't seem to be getting much better. He tells you that he really would rather not get tablets.

**Prescribe an antibiotic**: **Yes** ☐ **No** ☐

**On the scale 1 to 10, how difficult was it for you to make a decision for this scenario?**

| **Not at all** | 0 | 1 | 2 | 3 | 4 | 5 | 6 | 7 | 8 | 9 | 10 | **Extremely difficult** |
|---|---|---|---|---|---|---|---|---|---|---|---|---|

*If you wish to comment on this decision please do so here.*

**3.** The next patient is a 30-year-old woman. This is her third visit to the surgery with URTI symptoms in as many months; she is worried about the amount of time she has taken off work over this period. She tells you she has not been right since the really bad chest infection she had, and that she still is occasionally feeling her temperature rise at night. She tells you she would like something to make her better.

**Prescribe an antibiotic:** **Yes** ☐ **No** ☐

**On the scale 1 to 10, how difficult was it for you to make a decision for this scenario?**

| **Not at all** | 0 | 1 | 2 | 3 | 4 | 5 | 6 | 7 | 8 | 9 | 10 | **Extremely difficult** |
|---|---|---|---|---|---|---|---|---|---|---|---|---|

*If you wish to comment on this decision please do so here.*

**4.** A 38-year-old mother of 3 and smoker comes in next. Your partner saw her 4 days ago. For the past few days she has been experiencing a "deep chesty cough" and bringing up purulent sputum. Your partner advised rest, fluids and painkillers, but the symptoms are still persisting. She tells you that she feels that the problem has gone on long enough for her not to be "fobbed off" with more painkillers.

**Prescribe an antibiotic: Yes** ☐ **No** ☐

**On the scale 1 to 10, how difficult was it for you to make a decision for this scenario?**

| | | | | | | | | | | | | |
|---|---|---|---|---|---|---|---|---|---|---|---|---|
| **Not at all** | 0 | 1 | 2 | 3 | 4 | 5 | 6 | 7 | 8 | 9 | 10 | **Extremely difficult** |

*If you wish to comment on this decision please do so here.*

**5.** The next patient is a 63-year-old woman she has had a sore throat and runny nose for three days. It is 4.45pm on Friday and your appointments are running 20 minutes late. On examination she has a red pharynx. She states that she won't take up much of your time. All she wants is the penicillin she was given by your partner earlier this year for the same thing. You see from her notes that she is a frequent attender.

**Prescribe an antibiotic: Yes** ☐ **No** ☐

**On the scale 1 to 10, how difficult was it for you to make a decision for this scenario?**

| | | | | | | | | | | | | |
|---|---|---|---|---|---|---|---|---|---|---|---|---|
| **Not at all** | 0 | 1 | 2 | 3 | 4 | 5 | 6 | 7 | 8 | 9 | 10 | **Extremely difficult** |

*If you wish to comment on this decision please do so here.*

**6.** The next patient is a 22-year-old man. He is about to go on holiday in two days. He says he has been experiencing a sore throat for a few days. He has been taking painkillers, which he thinks provide some relief. His mother made him come today as she's worried it will affect his asthma, which he tells you has been playing up recently. He just doesn't want to miss out on his holiday.

**Prescribe an antibiotic: Yes** ☐ **No** ☐

**On the scale 1 to 10, how difficult was it for you to make a decision for this scenario?**

| | | | | | | | | | | | | |
|---|---|---|---|---|---|---|---|---|---|---|---|---|
| **Not at all** | 0 | 1 | 2 | 3 | 4 | 5 | 6 | 7 | 8 | 9 | 10 | **Extremely difficult** |

*If you wish to comment on this decision please do so here.*

## SECTION 5: YOUR GENERAL STYLE

| **In general, how true are the following statements about you?** | *Not at all true* | *Barely true* | *Moderately true* | *Exactly true* |
|---|---|---|---|---|
| a) I can always manage to solve difficult problems if I try hard enough | 1 | 2 | 3 | 4 |
| b) If someone opposes me, I can find means and ways to get what I want | 1 | 2 | 3 | 4 |
| c) It is easy for me to stick to my aims and accomplish my goals | 1 | 2 | 3 | 4 |
| d) I am confident that I could deal efficiently with unexpected events | 1 | 2 | 3 | 4 |
| e) Thanks to my resourcefulness, I know how to handle unforeseen situations | 1 | 2 | 3 | 4 |
| f) I can solve most problems if I invest the necessary effort | 1 | 2 | 3 | 4 |
| g) I can remain calm when facing difficulties because I can rely on my coping abilities | 1 | 2 | 3 | 4 |
| h) When I am confronted with a problem, I can usually find several solutions | 1 | 2 | 3 | 4 |
| i) If I am in a bind, I can usually think of something to do | 1 | 2 | 3 | 4 |
| j) No matter what comes my way, I'm usually able to handle it | 1 | 2 | 3 | 4 |

*This page will be removed and stored separately from the remainder of the questionnaire to preserve confidentiality*

☐ ☐☐☐

# *Background*

**Please check that your contact details are correct and amend if necessary.**

**Your Contact Phone Number** ____________________ **e-mail** ____________________

**B1. Male** ☐ **Female** ☐

**B2. How long have you been qualified as a doctor?** ☐☐ **Years**

**B3. How many full time equivalent principals (excluding you) are there in your practice?** ☐☐

**B4. Are you a GP trainer in a vocational training scheme?** **Yes** ☐ **No** ☐

**B5. How many clinical sessions (0.5 days) do you work per week on average?** ☐☐

**B6. What is your approximate total practice list size?** ☐☐☐☐☐☐

**B7. Which computer system does your practice use? Please indicate below**

| | **Computer system** | **Please tick if yes** |
|---|---|---|
| 1. | GPASS | |
| 2. | Emis | |
| 3. | Other (*please specify*) | |

**B8. Over the last six months how often have acute antibiotic prescriptions been written/printed by someone else (e.g. locum/trainee) using your cipher number? (please tick one option)**

**Never** ☐ **Sometimes** ☐ **Frequently** ☐ **Don't know** ☐

# Participation Slip

## Please answer the following question:

I authorise the Information & Statistics Division Primary Care Information Group, to release my prescribing data (BNF chapter 5) between September 2003 and October 2004

**Please tick the appropriate box**

| Yes | No |
|---|---|
| | |

Signature…………………………. Date …../…../……..

*Please return the completed questionnaire and consent form in the enclosed pre-paid envelope.*
*We would like to thank you for your participation in this study - your contribution is very much appreciated.*
